# NCOA5 promotes proliferation, migration and invasion of colorectal cancer cells via activation of PI3K/AKT pathway

**DOI:** 10.18632/oncotarget.22429

**Published:** 2017-11-14

**Authors:** Kailv Sun, Sheng Wang, Jun He, Yufeng Xie, Yang He, Zhenxin Wang, Lei Qin

**Affiliations:** ^1^ Department of General Surgery, The First Affiliated Hospital of Soochow University, Suzhou, China; ^2^ Department of Oncology, The First Affiliated Hospital of Soochow University, Suzhou, China; ^3^ Ministry of Health Key Laboratory of Thrombosis and Hemostasis, Jiangsu Institute of Hematology, The First Affiliated Hospital of Soochow University, Suzhou, China; ^4^ Collaborative Innovation Center of Hematology, Soochow University, Suzhou, China

**Keywords:** nuclear receptor coactivator 5 (NCOA5), colorectal cancer (CRC), proliferation, metastasis, phosphatidylinositol 3 kinase/protein kinase B (PI3K/AKT) pathway

## Abstract

The nuclear receptor coactivator 5 (NCOA5) displays both coactivator and corepressor functions. Previous studies showed that alteration of NCOA5 participates in carcinogenesis and progression. However, its roles in colorectal cancer (CRC) remain unknown. Herein, we demonstrated that expression of NCOA5 in human CRC tissues was notably higher than that in adjacent tissues, which significantly correlated with clinicopathological features such as length of tumor, regional lymph node staging and cancer staging. Knockdown of NCOA5 markedly suppressed proliferation, migration and invasion of SW620 high malignant CRC cells. Silencing of NCOA5 also inhibited *in vivo* growth of SW620 CRC subcutaneously xenografted tumors in athymic BALB/c nude mice. Meanwhile, Overexpression of NCOA5 facilitated these processes in SW480 low malignant CRC cells. Furthermore, knockdown of NCOA5 induced cell cycle G1 phase arrest in SW620 cells, whereas overexpression of NCOA5 promoted G1 to S phase transition in SW480 cells. Mechanistic studies revealed that NCOA5 upregulated phospho-protein kinase B (p-PKB/AKT), Cyclin D1 and matrix metalloproteinase 9 (MMP9) as well as downregulated P27 in CRC cells. Notably, PI3K inhibitor LY294002 obviously attenuated the effects of NCOA5 on p-AKT, Cyclin D1, P27 and MMP9. Moreover, LY294002 and knockdown of Cyclin D1 or MMP9 remarkably blocked the tumor-promoting activity of NCOA5. Collectively, NCOA5 promoted CRC cell proliferation, migration and invasion by upregulating Cyclin D1 and MMP9 while downregulating P27 to a great extent via activating PI3K/AKT signaling pathway. These findings suggested that NCOA5 exhibits an oncogenic effect in human CRC and represents a novel therapeutic target for CRC.

## INTRODUCTION

Colorectal cancer (CRC) is the second most commonly diagnosed malignancy in women and the third in men worldwide, which is one of the leading causes of cancer-related mortality [1]. Approximately 1.4 million new cases and 693,900 deaths occurred in global in 2012 [1]. The prognosis and overall survival of CRC still remains poor due to late diagnosis and metastasis [2]. Accumulating alteration of oncogenes and tumor suppressor genes as well as dysregulated signaling pathways are crucial for tumorgenesis and progression of CRC. Although recent advances in molecular biology have led to an increased knowledge of mechanisms responsible for CRC, only a limited number of critical molecules those have clinicopathological significance in CRC have been discovered. Therefore, seeking other master genes involved in CRC progression may help to provide more reliable therapeutic targets and improve prognosis of CRC.

The nuclear receptor coactivator 5 (NCOA5), also known as coactivator independent of AF2 (CIA), is a coregulator for α and β estrogen receptors and RVR (NR1D2) orphan nuclear receptor [3]. NCOA5 contains both LxxLL coactivator and ФxxФФ corepressor motifs, which exerts coactivator and corepressor effects [3–6]. Functionally, NCOA5 can interact with ERα and ERβ, thus enhancing their transcription activities [3, 4]. In addition, NCOA5 as a corepressor can cooperate with TIP30 tumor suppressor and subsequently inhibit ERα-induced *c-myc* transcription [5]. NCOA5 also functions as an LXR corepressor to attenuate expression of ATP-binding cassette subfamily A member 1 (ABCA1) [6]. Interestingly, a previous study [7, 8] revealed that NCOA5 insufficiency increases the risk of both glucose intolerance and inflammatory phenotype, resulting in the development of hepatocellular carcinoma (HCC). Furthermore, reduced NCOA5 expression is frequently found in HCC [7]. Other research [9] also found that NCOA5 is decreased in esophageal squamous cell carcinoma (ESCC), which is associated with its progression and a potential biomarker in predicting poor prognosis. Conversely, NCOA5 has been found to be upregulated in luminal breast cancer and associated with lower overall survival [10]. These reports indicated that alteration of NCOA5 contributes to carcinogenesis and cancer progression.

However, the roles of NCOA5 in human cancers are largely unknown. The expression pattern and biological effects of NCOA5 in CRC have not been reported. In the present study, we thus detected the expression of NCOA5 in human CRC clinical tissues and cell lines, and then analyzed the relationship between NCOA5 expression in CRC and its clinical implication. Furthermore, we investigated the effects of NCOA5 on CRC cell proliferation, migration and invasion *in vitro* and CRC subcutaneously (s.c.) xenografted tumor growth *in vivo* by lentivirus-mediated NCOA5 knockdown and overexpression, and also elucidated the potential molecular mechanisms.

## RESULTS

### NCOA5 is upregulated in CRC clinical samples and correlated with clinicopathological features of CRC

**Figure 1:**
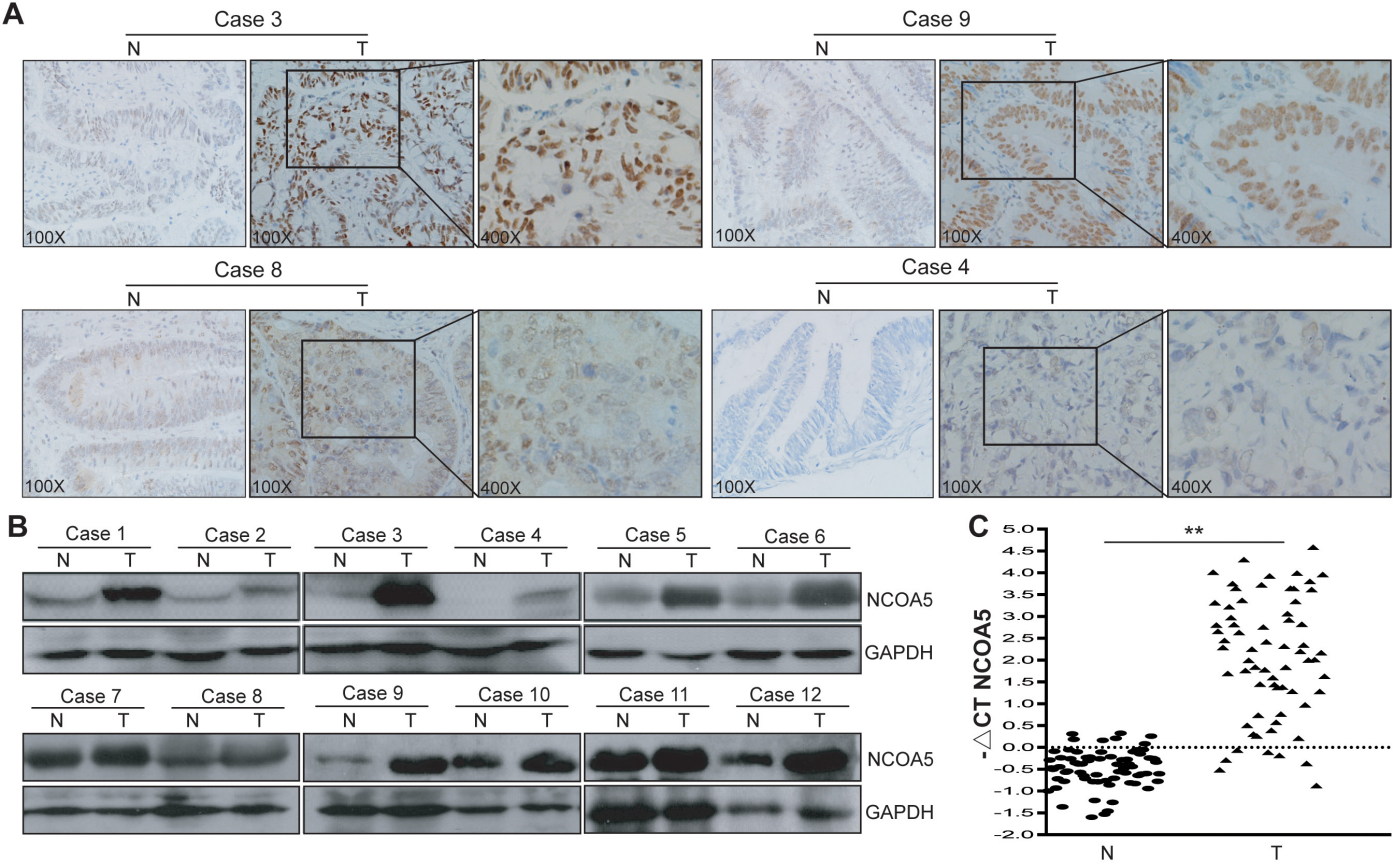
NCOA5 is highly expressed in CRC clinical tissues **(A)** Immunohistochemistry analysis of NCOA5 in CRC tissue specimens. The representative pictures (Case 3, +++; Case 9, ++; Case 8, +; and Case 4, -) of immunohistochemical staining were shown. T, colorectal tumor tissue; and N, adjacent non-tumor normal tissue. **(B)** Western blot analysis of NCOA5 in CRC tissue specimens. The representative pictures of Western blot analysis of NCOA5 in 12 pairs of T and N tissues derived from 12 CRC cases were shown. GAPDH was used as a loading control. **(C)** Real-time RT-PCR analysis of NCOA5. The RNAs derived from 70 pairs of CRC T and N tissues were subjected to SYBR Green I-based real-time quantitative PCR analysis of transcriptional expression of NCOA5. The level of NCOA5 mRNA was expressed as −ΔCT_NCOA5_=−(mean CT_NCOA5_−mean CT_GAPDH_). ^**^, *P*<0.01 compared with N tissue, Student *t* test, n=3 replicates per sample. Data shown were representative of two independent experiments.

To characterize its expression pattern in CRC, the expression of NCOA5 in human CRC tumor tissues and adjacent non-cancerous normal tissues was evaluated by immunohistochemistry analysis (Figure 1A). Immunohistochemical NCOA5 expression was available in all the 70 CRC cases. Among these CRC tumor tissue samples, fifty-five cases (78.6%) showed high expression of NCOA5 (32 samples scored “+++”, and 23 samples scored “++”) and fifteen cases showed low expression of NCOA5 (13 samples scored “+”, and 2 samples scored “-”). In contrast, NCOA5 was markedly decreased or not detected in the adjacent non-cancerous tissues. The expression of NCOA5 in human CRC clinical tissues was further confirmed by Western blot (Figure 1B) and qRT-PCR (Figure 1C) (*P*<0.01) analysis. To assess the clinical significance of NCOA5 in CRC, the relationship between NCOA5 expression and clinicopathological features was investigated. As shown in Table 1, high expression of NCOA5 was notably associated with the length of tumor (*P*<0.001), regional lymph node staging (*P*=0.005) and cancer staging (*P*=0.018), suggesting that NCOA5 positively modulates malignant progression of CRC. Although no statistical significance (*P*=0.145) was found, well-differentiated tumor was less frequently occurred in the high-expression group than that occurred in the low-expression group (Table 1).

**Table 1:** The relationship of NCOA5 expression with CRC clinicopathological features (Pearson's χ^2^ test)

Variables	Total	NCOA5		*P*
	n=70	Lown=15(21.4%)	Highn=55(78.6%)	
Gender				0.552
Male	42(60.0%)	10	32	
Female	28(40.0%)	5	23	
Age (year)				0.667
< 65	34(48.6%)	8	26	
≥ 65	36(51.4%)	7	29	
Length of tumor (cm)				< 0.001
< 4	25(35.7%)	12	13	
≥ 4	45(64.3%)	3	41	
Differentiation				0.145
Well	7(10.0%)	3	4	
Moderate/Poor	63(90.0%)	12	51	
Tumor location				0.506
Colon	47(67.1%)	9	38	
Rectum	23(32.9%)	6	17	
Depth of infiltrations				0.276
T1/T2/T3	16(22.9%)	5	11	
T4	54(77.1%)	10	44	
Regional Lymph nodes				0.005
N0	38(54.3%)	13	25	
N1/N2/N3	32(45.7%)	2	30	
Stage				0.018
I/II	37(52.9%)	12	25	
III/IV	33(47.1%)	3	30	

### Lentivirus-mediated NCOA5 knockdown and overexpression in CRC cells

**Figure 2:**
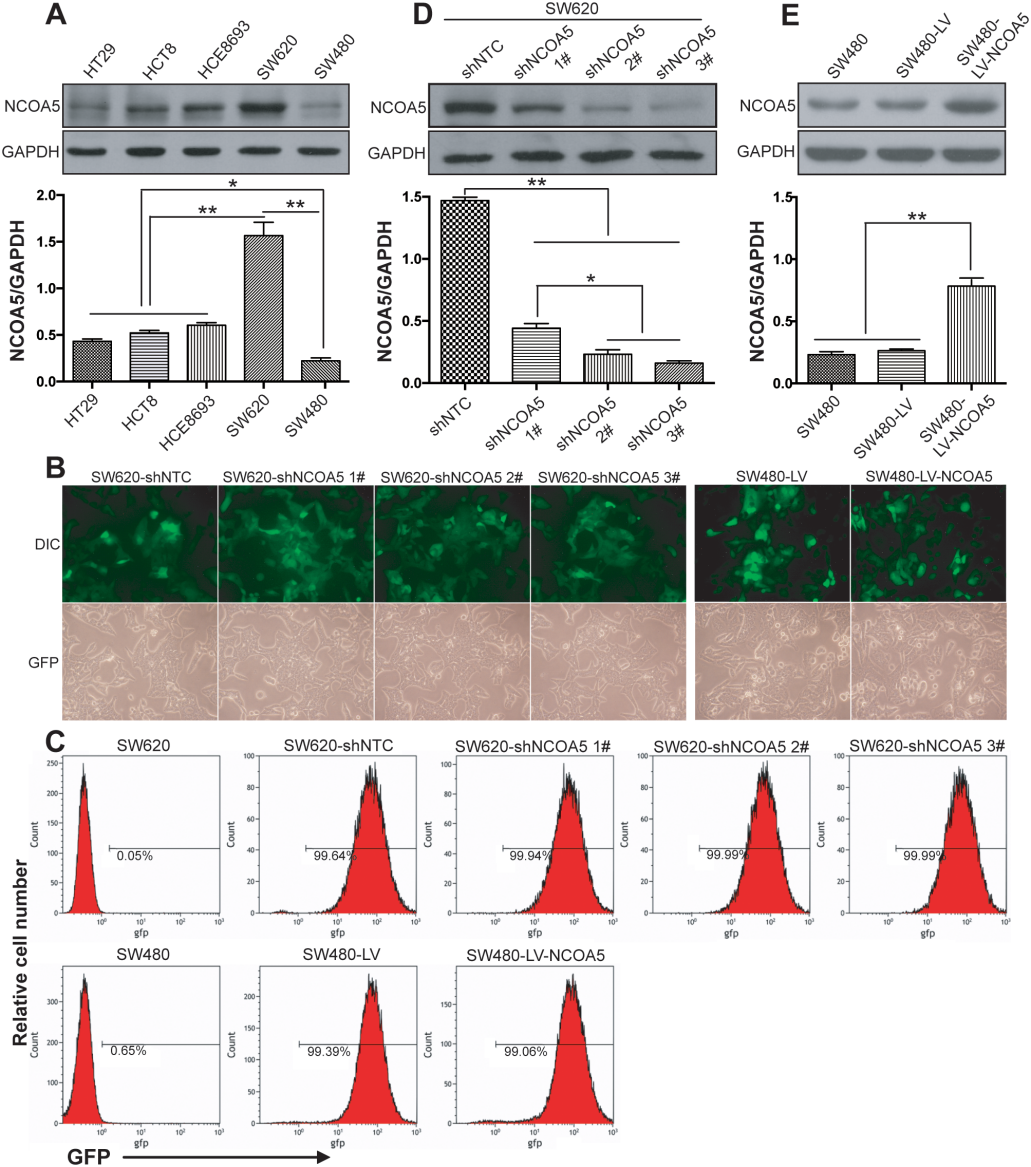
Lentivirus-mediated knockdown and overexpression of NCOA5 in CRC cells **(A)** Western blot analysis of NCOA5 in a panel of CRC cell lines. The lysates of HT29, HCT8, HCE8693, SW620 and SW480 CRC cells were immunoblotted with anti-NCOA5 or anti-GAPDH (a loading control) antibody. ^**^, *P*<0.01 compared withHT29, HCT8, HCE8693 and SW480 group; ^*^, *P*<0.05 compared withHT29, HCT8 and HCE8693 group, one-way repeated measures ANOVA, n=3 replicates per sample. **(B)** Fluorescence microscopic analysis of GFP expression. The GFP expression in NCOA5-silenced SW620 and NCOA5-overexpressed SW480 CRC cells was examined under fluorescence microscopy. Representative pictures of GFP and differential interference contrast (DIC) were shown. **(C)** Flow cytometric analysis of GFP expression. **(D)** Western blot analysis of lentivirus-mediated NCOA5 knockdown in SW620 CRC cells. The lysates of SW620-shNCOA5 1^#^, SW620-shNCOA5 2^#^, SW620-shNCOA5 3^#^ and SW620-shNTC CRC cells were immunoblotted with anti-NCOA5 or anti-GAPDH (a loading control) antibody. ^**^, *P*<0.01 compared withSW620-shNTC group; ^*^, *P*<0.05 compared withSW620-shNCOA5 1^#^group, one-way repeated measures ANOVA, n=3 replicates per sample. **(E)** Western blot analysis of lentivirus-mediated NCOA5 expression in SW480 cells. The lysates of SW480-LV-NCOA5, SW480-LV and SW480 CRC cells were immunoblotted with anti-NCOA5 or anti-GAPDH (a loading control) antibody. ^**^, *P*<0.01 compared withSW480-LV and SW480 group, one-way repeated measures ANOVA, n=3 replicates per sample. The expression level of NCOA5 in these Western blot assays was normalized to GAPDH and expressed as a NCOA5/GAPDH ratio. Data shown were representative of three independent experiments.

We firstly determined the expression level of NCOA5 in five different human CRC cell lines (HT29, HCT8, HCE8693, SW620 and SW480). Among them, SW620 is a highly malignant and metastatic CRC cell line which derived from metastases. As shown in Figure 2A, the highest expression of NCOA5 was found in SW620 cells (*P*<0.01), whereas the lowest expression of NCOA5 was found in SW480 cells (*P*<0.05 or 0.01). Thus, we selected SW620 and SW480 CRC cells for our next loss-of-function and gain-of-function researches by lentivirus-mediated NCOA5 shRNA knockdown and overexpression, respectively. Fluorescence microscopic analysis (Figure 2B) showed that almost all of the NCOA5 shRNA- or NCOA5-expressing lentivirus-transduced and corresponding control lentivirus-transduced SW620 or SW480 CRC cells exhibited GFP expression. Flow cytometric analysis (Figure 2C) further quantitatively demonstrated more than 90% GFP-positive cells in the above lentivirus-transduced SW620 or SW480 cells. The lentivirus-mediated knockdown or overexpression of NCOA5 in SW620 or SW480 CRC cells was then analyzed by Western blot. As shown in Figure 2D, SW620 cells displayed a drastic reduction in the expression of NCOA5 after transduced by NCOA5 shRNAs (shNCOA5 1#, shNCOA5 2# and shNCOA5 3#) compared with that transduced by shNTC (*P*<0.01). Moreover, the silencing efficiency of shNCOA5 2# and shNCOA5 3# was up to around 90%, which were much more efficient than shNCOA5 1# (Figure 2D) (*P*<0.05). Therefore, we employed SW620-shNCOA5 2# and SW620-shNCOA5 3# cell models for the following NCOA5 loss-of-function studies. In addition, SW480 cells showed an obvious increase in NCOA5 after lentivirus-directed NCOA5 gene transfer (Figure 2E) (*P*<0.01). Our results revealed that lentivirus-mediated NCOA5-silenced SW620 and NCOA5-transgenic SW480 CRC cell lines were generated.

### Knockdown of NCOA5 inhibits CRC cell proliferation and induces cell cycle G1 phase arrest, whereas overexpression of NCOA5 enhances its proliferation and G1 to S phase transition

**Figure 3:**
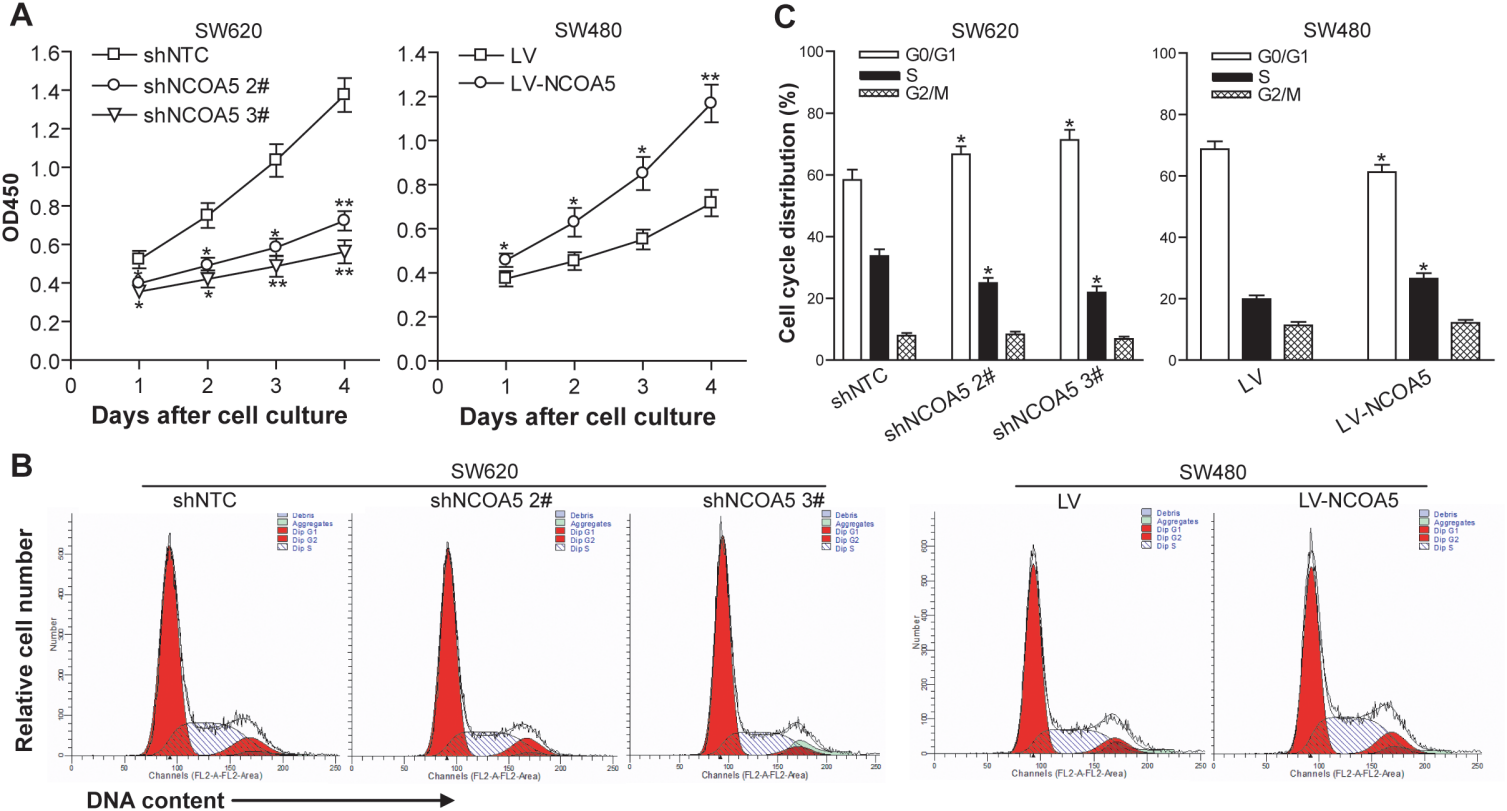
NCOA5 promotes CRC cell proliferation and G1 to S phase transition **(A)** CCK-8 assay. The SW620-shNCOA5 2^#^, SW620-shNCOA5 3^#^ and SW620-shNTC; SW480-LV-NCOA5 and SW480-LV CRC cells were cultured and the cell proliferation ability was evaluated by CCK-8 assay at the indicated culture time points. SW620-shNCOA5 2^#^ compared with SW620-shNTC group: ^*^, *P*<0.05 at day 1, 2 and 3, and ^**^, *P*<0.01 at day 4; SW620-shNCOA5 3^#^ compared with SW620-shNTC group: ^*^, *P*<0.05 at day 1 and 2, and ^**^, *P*<0.01 at day 3 and 4; SW480-LV-NCOA5 compared with SW480-LV group: ^*^, *P*<0.05 at day 1, 2 and 3, and ^**^, *P*<0.01 at day 4, two-way repeated measures ANOVA, n=6 replicates per condition.**(B** and **C)** Flow cytometric analysis of cell cycle profile. The cell cycle of SW620-shNCOA5 2^#^, SW620-shNCOA5 3^#^ and SW620-shNTC; SW480-LV-NCOA5 and SW480-LV CRC cells was analyzed using PI staining by flow cytometry. The representative flow cytometric figures were shown (B). The percentage of each cell cycle (G0/G1, S and G2/M phase) distribution in the total cell population was presented (C). SW620: ^*^, *P*<0.05 compared with SW620-shNTC group, one-way repeated measures ANOVA; SW480: ^*^, *P*<0.05 compared with LV group, Student *t* test, n=3 replicates per condition, n=2 replicates per sample. Data shown were representative of four independent experiments.

To investigate the biological role of NCOA5 in CRC cell growth, the *in vitro* proliferation ability of NCOA5-silenced SW620 and NCOA5-overexpressed SW480 tumor cells was determined by a CCK-8 assay. As shown in Figure 3A, the growth curves in the SW620-shNCOA5 2# and SW620-shNCOA5 3# groups were much lower than that in the shNTC-transduced control group (SW620-shNCOA5 2#, *P*<0.05 at day 1, 2 and 3, and *P*<0.01 at day 4; SW620-shNCOA5 3#, *P*<0.05 at day 1 and 2, and *P*<0.01 at day 3 and 4), showing that lentivirus-mediated NCOA5 knockdown obviously suppresses SW620 CRC cell growth in a time-dependent manner. Conversely, forced expression of NCOA5 significantly increased SW480 CRC cell proliferation compared with the LV-transduced control group (Figure 3A) (*P*<0.05 at day 1, 2 and 3, and *P*<0.01 at day 4). These results indicated that NCOA5 has a strong promoting effect on CRC cell proliferation. To examine whether NCOA5 facilitates CRC cell proliferation through directly accelerating cell cycle progression, flow cytometric analysis was applied to detect the cell cycle distribution in shNCOA5 2#- or shNCOA5 3#-transduced vs shNTC-transduced SW620 cells, and LV-NCOA5-transduced vs LV-transduced SW480 cells. As shown in Figure 3B and 3C, knockdown of NCOA5 by shNCOA5 2# or shNCOA5 3# in SW620 cells induced cell cycle G1 phase arrest and resulted in an accumulation of cells on G0/G1 phase from 58.40% to 66.67% or 71.32% (*P*<0.05) as well as a reduction of cells on S phase from 33.75% to 24.97 or 21.85% (*P*<0.05), whereas overexpression of NCOA5 in SW480 cells increased the entry of G1 to S phase (G0/G1, *P*<0.05, and S, *P*<0.05). Our data indicated that NCOA5 promotes CRC cell proliferation and growth very probably via stimulating G1 to S phase transition.

### Knockdown of NCOA5 represses CRC cell growth *in vivo*, whereas overexpression of NCOA5 facilitates its growth

**Figure 4:**
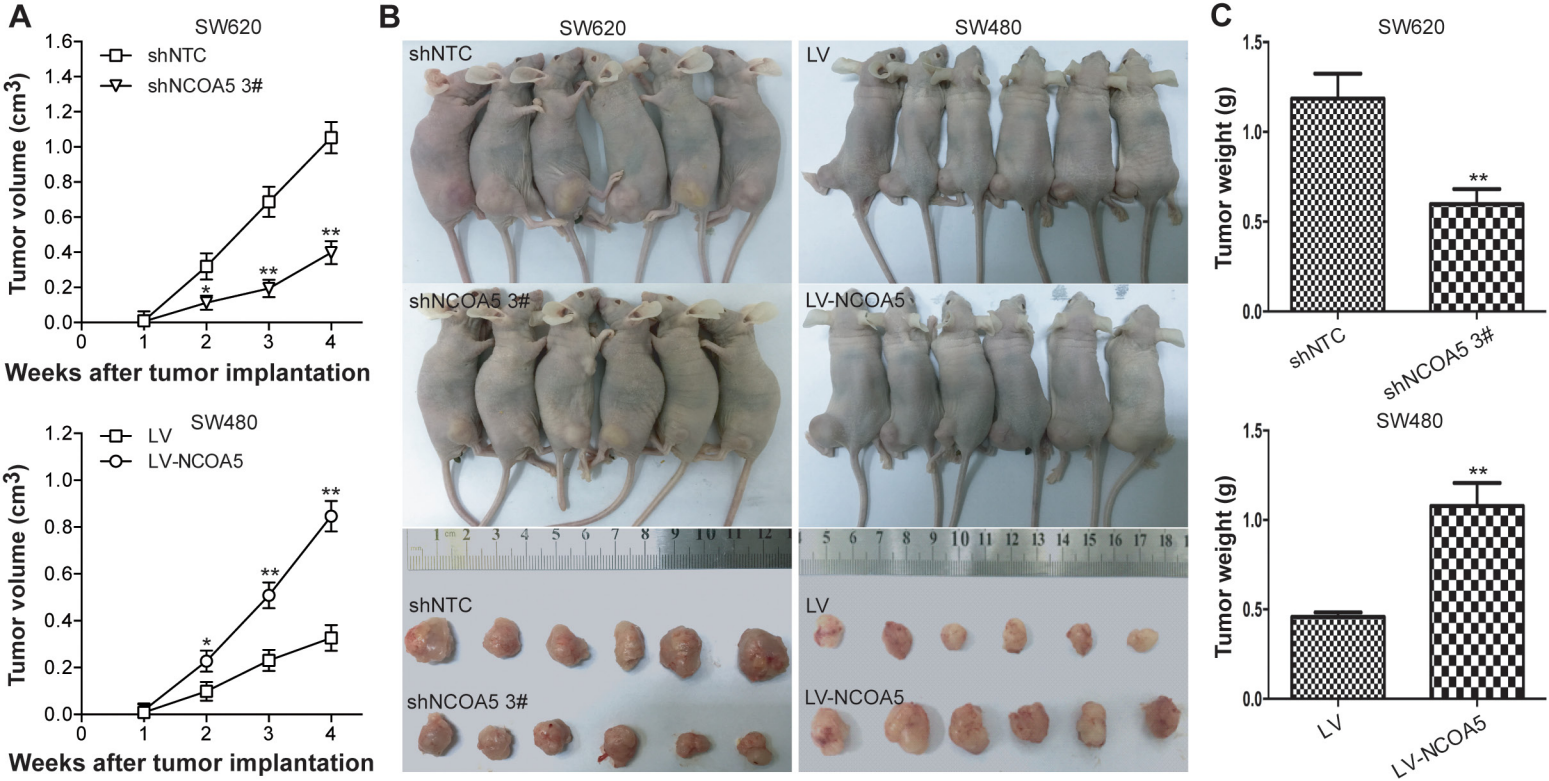
Knockdown of NCOA5 inhibits CRC xenografted tumor growth, whereas overexpression of NCOA5 promotes its growth The SW620-shNCOA5 3^#^ and SW620-shNTC; SW480-LV-NCOA5 and SW480-LV CRC cells s.c. injected into nude mice. The tumor volume **(A)** was measured after implantation of tumor cells.SW620-shNCOA5 3^#^ compared with SW620-shNTC group: ^*^, *P*<0.05 at week 2, and ^**^, *P*<0.01 at week 3 and 4; SW480-LV-NCOA5 compared with SW480-LV group: ^*^, *P*<0.05 at week 2, and ^**^, *P*<0.01 at week 3 and 4, two-way repeated measures ANOVA, n=6 replicates per condition. The xenografted tumors were removed **(B)** 4 weeks after tumor cell's implantation and tumor weight **(C)** was then measured. SW620-shNCOA5 3^#^ compared with SW620-shNTC group: ^**^, *P*<0.01; SW480-LV-NCOA5 compared with SW480-LV group: ^**^, *P*<0.01, Student *t* test, n=6 replicates per condition. Data shown were representative of three independent experiments.

To further assess whether the effect of NCOA5 on *in vitro* growth of CRC cells could be reproduced *in vivo*, we monitored and compared human CRC s.c. xenografted tumor growth of SW620-shNCOA5 3# vs SW620-shNTC, and SW480-LV-NCOA5 vs SW480-LV in athymic BALB/c nude mice. As shown in Figure 4, knockdown of NCOA5 by shNCOA5 3# remarkably retarded SW620 CRC cell growth *in vivo* (tumor volume, *P*<0.05 at week 2, and *P*<0.01 at week 3 and 4; tumor weight, *P*<0.01), whereas overexpression of NCOA5 promoted SW480 CRC cell growth (tumor volume, *P*<0.05 at week 2, and *P*<0.01 at week 3 and 4; tumor weight, *P*<0.01). Our data revealed that NCOA5 is also capable of promoting CRC cell growth *in vivo* and that NCOA5 may represent a potential therapeutic target for CRC.

### Knockdown of NCOA5 suppresses CRC cell migration and invasion, whereas overexpression of NCOA5 promotes these processes

**Figure 5:**
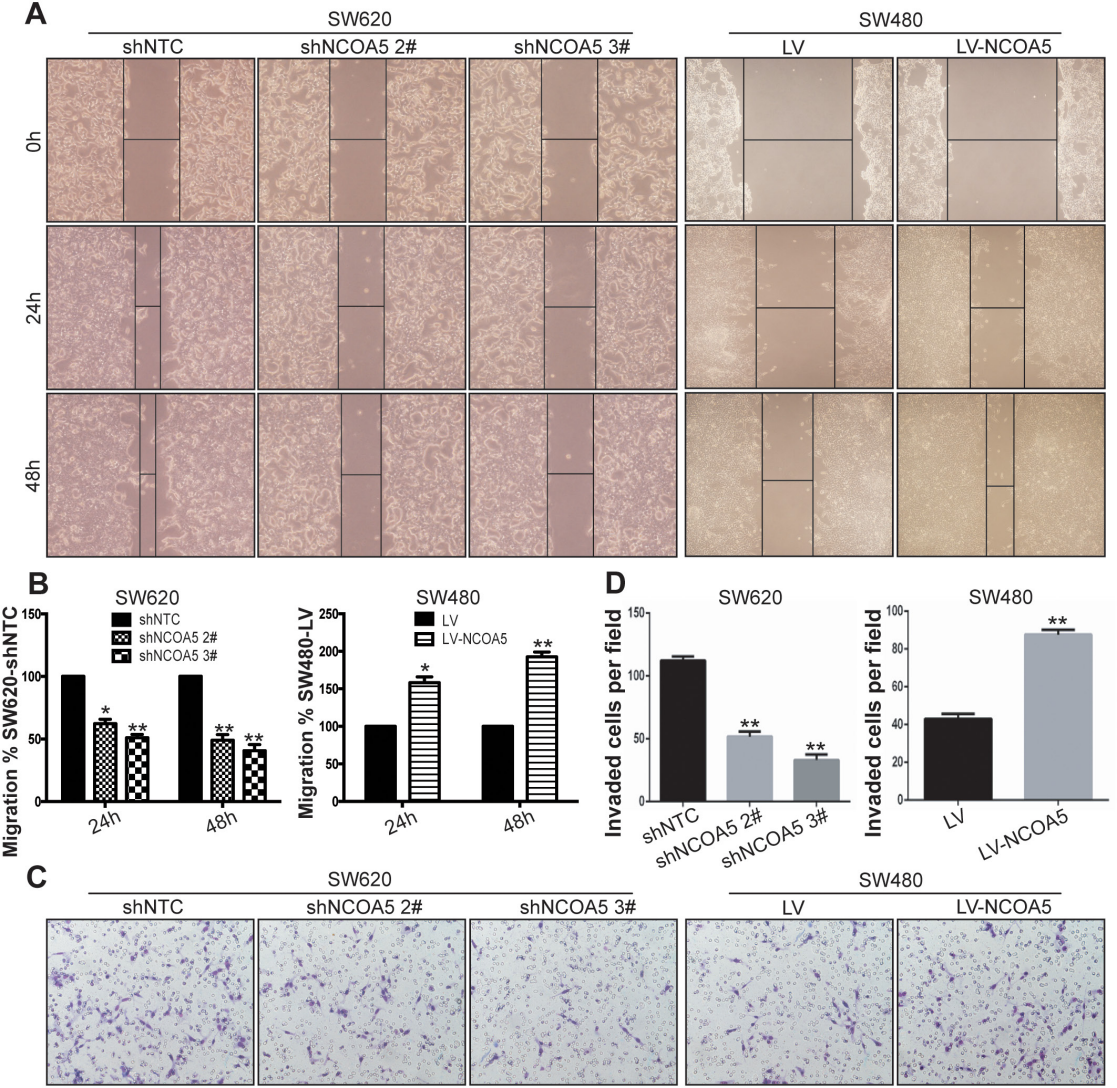
Knockdown of NCOA5 suppresses CRC cell migration and invasion, whereas forced expression of NCOA5 enhances these processes **(A** and **B)** Wound healing assay. The SW620-shNCOA5 2^#^, SW620-shNCOA5 3^#^ and SW620-shNTC; SW480-LV-NCOA5 and SW480-LV CRC cells were scratched in the monolayer with a pipette tip. Wound closures were photographed at 0, 24 and 48 hours after wounding. The representative figures of wound healing assay were shown (A). The relative migratory ability of control group was presented (B). SW620-shNCOA5 2^#^ compared with SW620-shNTC group: ^*^, *P*<0.05 at hour 24, and ^**^, *P*<0.01 at hour 48; SW620-shNCOA5 3^#^ compared with SW620-shNTC group: ^**^, *P*<0.01 at hour 24 and 48; SW480-LV-NCOA5 compared with SW480-LV group: ^*^, *P*<0.05 at hour 24, and ^**^, *P*<0.01 at hour 48, two-way repeated measures ANOVA, n=6 replicates per condition. **(C** and **D)** Transwell invasion assay. The representative figures of Transwell invasion assay were shown (C). The number of invaded tumor cells was counted (D). SW620-shNCOA5 2^#^ and SW620-shNCOA5 3^#^ compared with SW620-shNTC group: ^**^, *P*<0.01, one-way repeated measures ANOVA; SW480-LV-NCOA5 compared with SW480-LV group: ^**^, *P*<0.01, Student *t* test, n=3 replicates per condition, n=10 observations per replicate. Data shown were representative of four independent experiments.

To evaluate the association of NCOA5 with migration and invasion of CRC cells, wound healing assay and Transwell chamber invasion assay were conducted to examine the speed of wound closure and the potential of invasion in NCOA5-silenced SW620 and NCOA5-overexpressed SW480 CRC cells, respectively. The wound healing rate of SW620-shNCOA5 2# and SW620-shNCOA5 3# groups was dramatically lower than that of shNTC-transduced control group (Figure 5A and 5B) (SW620-shNCOA5 2#, *P*<0.05 at hour 24, and *P*<0.01 at hour 48; SW620-shNCOA5 3#, *P*<0.01 at hour 24 and 48). Lentivirus-directed NCOA5 overexpression facilitated the wound closure in SW480 cells compared to the LV-transduced control group (Figure 5A and 5B) (*P*<0.05 at hour 24, and *P*<0.01 at hour 48). Furthermore, the SW620-shNCOA5 2# and SW620-shNCOA5 3# groups showed a substantial reduction in invasive capacity, whereas SW480-LV-NCOA5 exhibited a marked enhancement in the process (Figure 5C and 5D) (*P*<0.01). These data suggested that NCOA5 promotes CRC cell motility and invasion.

### NCOA5 enhances the PI3K/AKT signaling pathway in CRC cells

**Figure 6:**
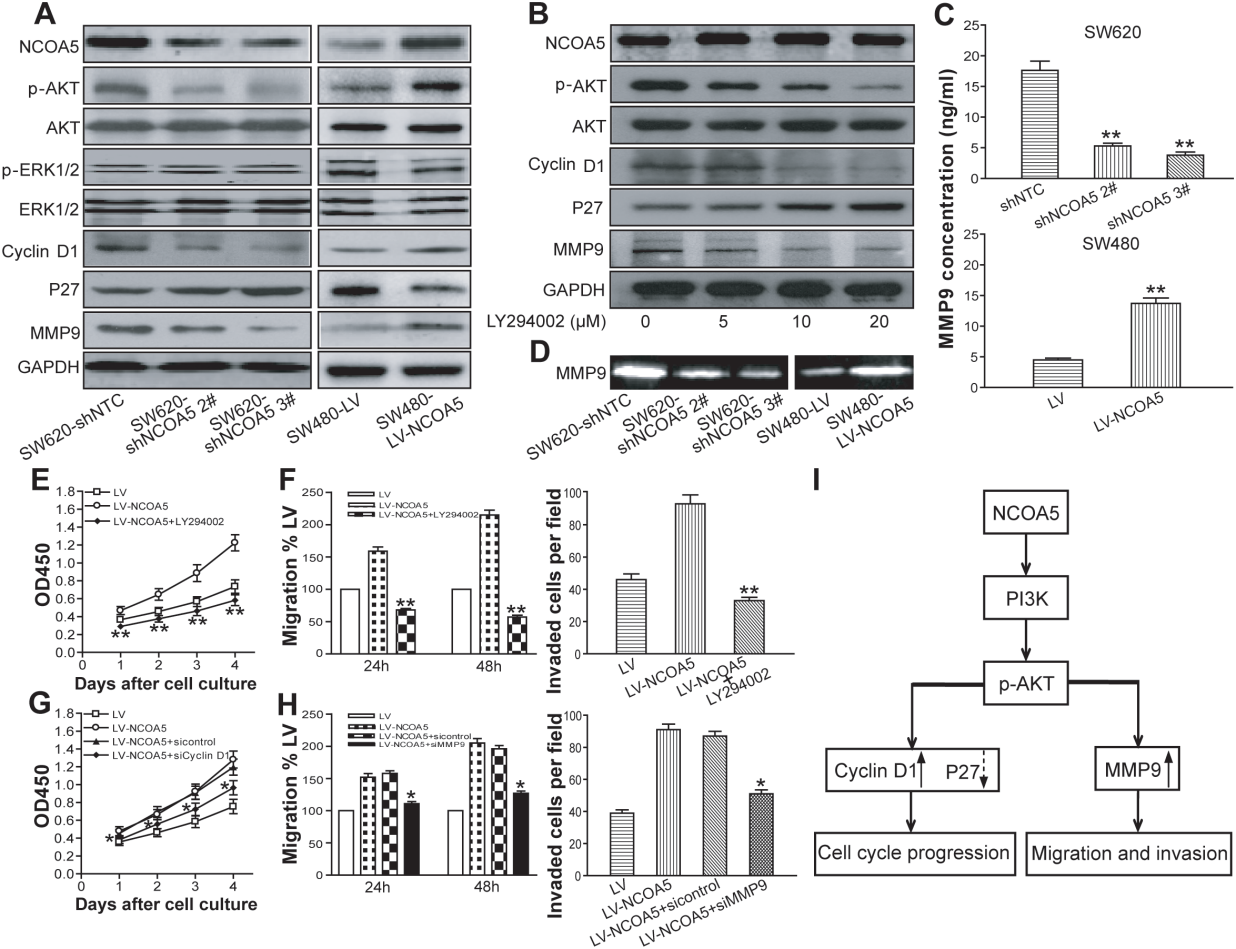
NCOA5 upregulates Cyclin D1 and MMP9 as well as downreuglates P27 in CRC cells via the PI3K/AKT pathway **(A)** Western blot analysis of NCOA5, p-AKT, AKT, p-ERK1/2, ERK1/2, Cyclin D1, P27 and MMP9. The lysates derived from SW620-shNCOA5 2^#^, SW620-shNCOA5 3^#^ and SW620-shNTC; SW480-LV-NCOA5 and SW480-LV CRC cells were immunoblotted with a panel of antibodies specific for NCOA5, p-AKT, AKT, p-ERK1/2, ERK1/2, Cyclin D1, P27, MMP9 and GAPDH (a loading control), respectively. The representative figures of Western blot assay were shown. **(B)** Western blot analysis after PI3K inhibition. The SW480-LV-NCOA5 CRC cells were pretreated with different concentrations (0, 5, 10 and 20 μM) of PI3K inhibitor LY294002. The lysates of LY294002-treated and -untreated SW480-LV-NCOA5 CRC cells were harvested and then immunoblotted with a panel of antibodies specific for NCOA5, p-AKT, AKT, Cyclin D1, P27, MMP9 and GAPDH (a loading control). The representative figures of Western blot assay were shown. **(C)** ELISA analysis of MMP9 secretion.SW620-shNCOA5 2^#^ and SW620-shNCOA5 3^#^ compared with SW620-shNTC group: ^**^, *P*<0.01, one-way repeated measures ANOVA; SW480-LV-NCOA5 compared with SW480-LV group: ^**^, *P*<0.01, Student *t* test, n=3 replicates per condition, n=3 replicates per sample. **(D)** Zymography analysis of MMP9 activity. The representative figures of gelatin zymography assay were shown. **(E)** CCK-8 assay after PI3K inhibition. ^**^, *P*<0.01 at day 1, 2, 3 and 4 compared with LY294002-untreated SW480-LV-NCOA5 group, two-way repeated measures ANOVA, n=6 replicates per condition. **(F)** Migration and invasion assays after PI3K inhibition. Migration: ^**^, *P*<0.01 at hour 24 and 48 compared with LY294002-untreated SW480-LV-NCOA5 group, two-way repeated measures ANOVA, n=6 replicates per condition; invasion: ^**^, *P*<0.01 compared with LY294002-untreated SW480-LV-NCOA5 group, one-way repeated measures ANOVA, n=3 replicates per condition, n=10 observations per replicate. **(G)** CCK-8 assay after Cyclin D1 siRNA knockdown. ^*^, *P*<0.05 at day 1, 2, 3 and 4 compared with SW480-LV-NCOA5 and siRNA control-transfected SW480-LV-NCOA5 groups, two-way repeated measures ANOVA, n=6 replicates per condition. **(H)** Migration and invasion assays after MMP9 siRNA knockdown. Migration: ^*^, *P*<0.05 at hour 24 and 48 compared with SW480-LV-NCOA5 and siRNA control-transfected SW480-LV-NCOA5 groups, two-way repeated measures ANOVA, n=6 replicates per condition; invasion: ^*^, *P*<0.05 compared with SW480-LV-NCOA5 and siRNA control-transfected SW480-LV-NCOA5 groups, one-way repeated measures ANOVA, n=3 replicates per condition, n=10 observations per replicate. **(I)** A schematic model of NCOA5's functions during CRC cell progression. Highly expressed NCOA5 promotes PI3K/AKT signaling pathway and consequently upregulates Cyclin D1 and MMP9 as well as downregulates P27, leading to CRC aggressive proliferation and metastasis cascade. Data shown were representative of three independent experiments.

To examine whether NCOA5 takes participation in proliferation, migration and invasion of CRC cells via PI3K/AKT [11–13] and MAPK/ERK [13, 14] pathways, the expression of related molecules such as phosphorylated AKT and ERK1/2 (p-AKT and p-ERK1/2) and total AKT and ERK1/2 in NCOA5-silenced SW620 and NCOA5-overexpressed SW480 CRC cells were assessed by Western blot analysis. As shown in Figure 6A, the expression of p-AKT in SW620-shNCOA5 2# and SW620-shNCOA5 3# groups was significantly lower than that in the shNTC-transduced control group. And the opposing effect was found in the SW480 cells when transduced by LV-NCOA5 (Figure 6A). However, NCOA5 exerted no effect on ERK1/2 activation (Figure 6A). Subsequently, we applied LY294002, a synthetic inhibitor of the p110 catalytic subunit of PI3K, to investigate the possible involvement of PI3K in NCOA5-mediated AKT activation in CRC cells. As expected, treatment of NCOA5-overexpressed SW480 cells with LY294002 significantly impaired NCOA5-induced AKT phosphorylation in a dose-dependent fashion, suggesting that enhancement of AKT activity in response to NCOA5 was PI3K dependent (Figure 6B). These results confirmed that NCOA5 activates the PI3K/AKT signaling pathway in human CRC cells.

### NCOA5 promotes CRC cell proliferation, migration and invasion by upregulation of Cyclin D1 and MMP9 as well as downregulation of P27 through PI3K/AKT pathway

To further explore molecular mechanisms responsible for NCOA5-mediated biological behavior in CRC, the expression of PI3K/AKT downstream molecules such as Cyclin D1, P27 and MMP9 [15–20] in NCOA5-silenced/overexpressed CRC cells was evaluated by Western blot analysis. We found that knockdown of NCOA5 decreased the expression of Cyclin D1 and MMP9 as well as increased the expression of P27 in SW620 cells, whereas overexpression of NCOA5 exerted opposing effects in SW480 cells (Figure 6A). To confirm the contribution of PI3K/AKT pathway to NCOA5-mediated effect on Cyclin D1, P27 and MMP9, the expression of Cyclin D1, P27 and MMP9 were further analyzed in LV-NCOA5-transduced SW480 cells after treatment with PI3K inhibitor LY294002. As shown in Figure 6B, LY294002 treatment decreased Cyclin D1 and MMP9 but increased P27 in the SW480-LV-NCOA5 cells compared with the LY294002-untreated SW480-LV-NCOA5 control group, indicating inhibition of PI3K/AKT activity obviously attenuated the regulatory effect of NCOA5 on Cyclin D1, P27 and MMP9. In addition, LY294002 exhibited no effect on the expression of NCOA5 (Figure 6B). Notably, knockdown of NCOA5 downregulated the secretion and the activity of MMP9 in SW620 cells, whereas overexpression of NCOA5 upregulated its secretion and activity in SW480 cells (Figure 6C and 6D) (*P*<0.01). To further validate the roles of PI3K/AKT signaling molecules in NCOA5-triggered functional effects in CRC, we performed assays with PI3K inhibition and downstream molecule Cyclin D1 or MMP9 siRNA knockdown in NCOA5-overexpressed SW480 CRC cells. As shown in Figure 6E (*P*<0.01 at day 1, 2, 3 and 4) and 6F (migration, *P*<0.01 at hour 24 and 48; invasion, *P*<0.01), LY294002 completely abolished the promoting effect of NCOA5 on CRC cell proliferation, migration and invasion. Moreover, knockdown of Cyclin D1 obviously impaired proproliferative activity of NCOA5 (Figure 6G) (*P*<0.05 at day 1, 2, 3 and 4). Additionally, knockdown of MMP9 also blunted the facilitating effect of NCOA5 on migration and invasion (Figure 6H) (*P*<0.05 at hour 24 and 48). Our data suggested that NCOA5 might promote proliferation, migration and invasion of CRC cells by upregulating Cyclin D1 and MMP9 as well as downregulating P27 to a large extent via activating PI3K/AKT pathway (Figure 6I).

## DISCUSSION

The present study provided the first compelling evidence that (i) NCOA5 is highly expressed in human CRC tissues; (ii) NCOA5 has a significant positive correlation with length of tumor, regional lymph node staging and cancer staging of CRC patients; and (iii) NCOA5 promotes proliferation, migration and invasion of CRC cells by upregulating Cyclin D1 and MMP9 as well as downregulating P27 through activating PI3K/AKT pathway. Accumulating evidence has shown that NCOA5 is downregulated in certain types of human cancers such as HCC and ESCC [7, 9]. More recently, NCOA5 has been found to be upregulated in luminal breast cancer [10], which is consistent with our report. These findings suggested that the antitumor or protumor effect of NCOA5 in human cancers may be dependent on tissue type.

Constitutive genomic studies demonstrated that PI3K/AKT signaling pathway is one of the most frequently deregulated pathways in CRC [21, 22]. PI3K phosphorylates AKT and consequently facilitates tumorigenesis and cancer progression through its downstream targets [11–13]. The G0/G1 phase markers such as Cyclin D1 and P27, downstream signaling molecules of PI3K/AKT pathway, are pivotal cell cycle regulators [15, 16]. Aberrant activation of PI3K/AKT pathway can enhance cancer cell proliferation via induction of Cyclin D1 and repression of P27 [17, 18, 23]. In this study, we demonstrated that NCOA5 upregulated p-AKT of CRC cells in a PI3K-dependent manner by Western blot and PI3K inhibitor assays, suggesting that NCOA5 is capable of augmenting PI3K/AKT activation in CRC cells. We also found that NCOA5 simultaneously increased Cyclin D1 and decreased P27 expression in CRC cells via PI3K/AKT pathway as evidenced by the fact that PI3K inhibitor significantly impaired the effects of NCOA5 on Cyclin D1 and P27. Furthermore, inhibition of PI3K or knockdown of Cyclin D1 remarkably attenuated NCOA5-induced proproliferative effect in CRC cells. Therefore, our data revealed that elevated expression of NCOA5 in CRC may result in deregulated cell cycle control to a great extent through the PI3K/AKT/Cyclin D1, P27 pathway, which may be a cause of CRC tumorigenesis and progression.

Cancer metastasis is a crucial factor for the poor prognosis of solid tumors and the major cause of cancer-associated deaths [24–26]. MMP9 as an important member of znic-dependent endopeptidases family has been considered to be involved in cancer invasion and metastasis via degradation of the extracellular matrix [20, 27]. Previous studies have revealed that PI3K/AKT/MMP9 pathway is closely associated with the process of cancer metastasis [19, 28, 29]. Previous studies have also shown that upregulated expression of MMP9 has an obvious relationship with advanced Dukes stage and distant metastasis in CRC [30, 31]. To decipher the molecular mechanism for NCOA5-mediated promoting effect in CRC cell migration and invasion, the expression and activity of PI3K/AKT downstream signaling molecule MMP9 in NCOA5-silenced/overexpressed CRC cells was further analyzed. We demonstrated that NCOA5 upregulated the expression, secretion and activity of MMP9 in CRC cells. Inhibition of PI3K activity markedly attenuated NCOA5-induced upregulation of MMP9, indicating that NCOA5 positively regulates MMP9 of CRC cells through the PI3K/AKT pathway. Functional assays further confirmed that either PI3K inhibitor or knockdown of MMP9 obviously blocked the facilitating effect of NCOA5 on CRC cell migration and invasion. Thus, these results validated that NCOA5 enhances migration and invasion of CRC cells very possibly via the PI3K/AKT/MMP9 pathway.

In conclusion, our study strongly showed that NCOA5 has an oncogenic effect in CRC. High expression of NCOA5 may be a valuable marker of CRC progression. NCOA5 promotes CRC cell proliferation, migration and invasion to a large extent via activation of PI3K/AKT/Cyclin D1, P27, MMP9 pathway. Our data revealed that NCOA5 may be used as a novel therapeutic target for CRC.

## MATERIALS AND METHODS

### CRC tissue samples and patients

A total of 70 pairs of snap-frozen or paraffin-embedded CRC and adjacent non-cancerous tissue samples were obtained from randomly selected 70 CRC patients who have undergone surgery at the First Affiliated Hospital of Soochow University, the Second Affiliated Hospital of Soochow University and the Second People's Hospital of Changshu from January 2010 to December 2014. The corresponding adjacent non-cancerous colorectal tissues were obtained at least 6 cm away from the tumor. The study protocol was approved by the Ethics Committee of the First Affiliated Hospital of Soochow University. All the participants signed the informed consent. Any patient received prior chemotherapy and/or radiotherapy was excluded. Pathological staging was reviewed independently by two experienced pathologists according to the 7th edition of the American Joint Committee's Cancer Staging Manual [32]. The clinicopathological characteristics of these patients were summarized based upon medical history.

### Immunohistochemistry (IHC) analysis

Formalin-fixed and paraffin-embedded CRC tissues and adjacent non-cancerous colorectal tissues were cut into 4 μm-thick sections. The sections were then deparaffinized and rehydrated. Antigen retrieval was performed by microaving the slides in 0.01 M citrate buffer (pH=6.0) for a total of 10 min. Endogenous peroxidase activity was quenched by treatment with 3% H_2_O_2_ for 30 min followed by incubation with goat serum for 15 min. Subsequently, the sections were incubated with rabbit anti-human NCOA5 (1:25; Cat. No. A300-789A, Bethyl, Montgomery, TX, USA) primary antibody in a humidity chamber overnight at 4 °C. The primary antibody was omitted for a negative control. Horseradish peroxidase (HRP)-labeled anti-rabbit secondary antibody was then incubated for 1 hour at room temperature and immunostaining signal was detected using a UltraSensitive^TM^ SP kit (Maxin, Fuzhou, Guangdong, China). Finally, the slides were counterstained with hematoxylin & eosin (HE) and coverslipped. IHC scoring was examined independently by two experienced histopathologists without knowledge of clinicopathological information. The percentage of positive tumor cells and the staining intensity were used to gain the IHC scoring. The percentage of positive tumor cells was assigned to 5 categories: ≤5% (0), 5-25% (1), 25-50% (2), 50-75% (3), and ≥75% (4). Positive cells (≤5%) were used as the cut-off to define negative tumors. The intensity of immunostaining was scored as follows: negative (0), weak (1), moderate (2), and strong (3). The percentage of positive tumor cells and staining intensity were added to produce a weighted score for each tumor specimen. The intensity scores were grouped as (-), 0-1; (+), 2-3; (++), 4-5; and (+++), 6-7. It was considered as high expression in tumor specimen when the final scores were ≥4 (++, +++) [33].

### Real-time RT-PCR analysis

The total RNAs of snap-frozen CRC tumor and paired non-cancerous tissues were extracted using a MiniBEST universal RNA extraction kit (TaKaRa, Dalian, Liaoning, China) and the purified RNAs were reversely transcribed to first-strand cDNAs by a RevertAid RT reverse transcription kit (Thermo Fisher Scientific, Waltham, MA, USA). The transcriptional expression of NCOA5 was then subjected to SYBR Green I-based real-time quantitative PCR analysis using a FastStart Universal SYBR Green Master (Rox) (Roche Applied Science, Penzberg, Upper Bavaria, Germany) following manufacturer's instructions. The human NCOA5- and GAPDH-specific primers (NCOA5-F, 5’-ATA CGG CTC CAT CAA GAC CC-3’ and NCOA5-R, 5’-TGG GCT CTC TCC TTG GAC TT-3’ for amplifying 114 bp; GAPDH-F, 5’-ACG GAT TTG GTC GTA TTG GG-3’ and GAPDH-R, 5’-CGC TCC TGG AAG ATG GTG AT-3’ for amplifying 214 bp) (Sangon Biotechnology Inc., Shanghai, China) was used. The authenticity of PCR products was verified by melting curve analysis and agarose gel electrophoresis. The relative expression level of NCOA5 mRNA was normalized to an internal control GAPDH and calculated by the ΔCT method ΔCT=(mean CT_NCOA5_−mean CT_GAPDH_) as previously described [34].

### Cell lines and cell culture

The human CRC cell lines such as HT29, HCT8, HCE8693, SW620 and SW480 were obtained from American Type Culture Collection (Manassas, VA, USA). The human embryonic kidney cell line 293T for lentivirus generation was purchased from the Cell Bank, Type Culture Collection of Chinese Academy of Sciences (Shanghai, China). All of the above-mentioned cells were grown in Dulbecco's modified Eagle's medium (DMEM) (HyClone, Logan, UT, USA) containing 10% fetal bovine serum (FBS) (Gibco-BRL, Gaithersburgh, MD, USA) and antibiotics (100 U/ml penicillin and 0.1 mg/ml streptomycin) (Sigma, St Louis, MO, USA).

### Lentivirus preparation and titration

To generate a lentivirus harboring short hairpin RNA (shRNA) targeting human NCOA5 (GenBank Accession No.: NM_020967), shNCOA5 1# (5’-GGA GAC AGT CGA GAT TCA AGG-3’), shNCOA5 2# (5’-GCG TAG AGA AGA GCT TTA TCG-3’) and shNCOA5 3# (5’-GCC AGA AAT TAT GAG CGT TAC-3’) (GenePharma, Shanghai, China) were designed and synthesized. A nontargeting control (5’-TTC TCC GAA CGT GTC ACG T-3’) was used as a shRNA control (shNTC). The shNCOA5 1#, shNCOA5 2#, shNCOA5 3# and shNTC were then inserted into a pGLVH1/GFP+Puro lentiviral plasmid (GenePharma, Shanghai, China) followed by cotransfection with pLP1, pLP2 and VSVG lentiviral packaging plasmids (GenePharma, Shanghai, China) into 293T cells using Lipofectamine 2000 (Thermo Fisher Scientific, Waltham, MA, USA), respectively. After 72 hours of transfection, the culture supernatants were harvested and the GFP-expressed lentiviruses including LV-shNCOA5 1#, LV-shNCOA5 2#, LV-shNCOA5 3# and LV-shNTC (used as a control) were then concentrated by ultracentrifugation (20000 rpm, 2 hours) as previously described [35]. To construct a lentivirus expressing human NCOA5, the coding sequence (CDS) of human NCOA5 was cloned from a human cDNA library. Then the full-length human NCOA5 CDS fragment was subcloned into a pLenti179-EF1a-PuroR-CMV-EGFP-3Flag lentiviral plasmid (Talen-Bio, Shanghai, China) to form a pLenti179-EF1a-PuroR-CMV-NCOA5-EGFP-3Flag recombinant lentiviral plasmid. The LV-NCOA5 expressing NCOA5 and EGFP, and LV expressing EGFP alone (used as a control) were subsequently produced by cotransfection and centrifugation similarly as described above, respectively. The biological titer (transducing unit/ml, abbreviated as TU/ml) of all lentiviruses was evaluated by calculating the number of GFP-expressing 293T cells after lentiviral infection under fluorescence microscopy. The ratio of infectious lentiviruses (TU) to target cells is called multiplicity of infection (MOI).

### Lentiviral infection and generation of stable cell lines

The SW620 CRC cells were infected with LV-shNCOA5 1#, LV-shNCOA5 2#, LV-shNCOA5 3# and LV-shNTC at a MOI of 20 plus 10 μg/ml of Polybrene (GeneChem, Shanghai, China), respectively. The SW480 CRC cells were infected with LV-NCOA5 and LV as described above. 72 hours after lentiviral infection, the tumor cells were selected with puromycin (Gibco-BRL, Gaithersburgh, MD, USA) at a final concentration of 2 μg/ml. The transgene efficiency of puromycin-resistant SW620 and SW480 cell derivatives including SW620-LV-shNCOA5 1# (SW620-shNCOA5 1#), SW620-LV-shNCOA5 2# (SW620-shNCOA5 2#), SW620-LV-shNCOA5 3# (SW620-shNCOA5 3#) and SW620-LV-shNTC (SW620-shNTC) (used as a control); SW480-LV-NCOA5 and SW480-LV (used as a control) was detected by fluorescence microscopic and flow cytometric analysis of GFP. The knockdown and overexpression efficiency of NCOA5 was further analyzed by Western blot.

### Cell proliferation assay

The NCOA5-silenced SW620 and NCOA5-overexpressed SW480 CRC cells and the corresponding control cells were seeded at a density of 4×10^3^ cells/200 μl culture medium per well in 96-well plates. Cell proliferation ability was analyzed 24, 48, 72 and 96 hours after cell culture using a Cell Counting Kit-8 (CCK-8) (Dojindo Molecular Technologies, Gaithersburg, MD, USA) according to the manufacturer's instructions. An automatic microplate reader measured the optical density (OD) of each well at 450 nm. Cell growth curves were plotted with OD value vs culture time.

### Cell cycle analysis

The NCOA5-silenced SW620 and NCOA5-overexpressed SW480 CRC cells and the corresponding control cells were cultured and collected. Then the cells were washed with phosphate-buffered saline (PBS) and fixed in ice-cold 70% ethanol at 4 °C overnight. They were subsequently washed with PBS, treated with 500 U/ml RNase A (Sigma, St Louis, MO, USA) at 37 °C for 30 min and stained with 50 μg/ml propidium iodide (PI) (Sigma, St Louis, MO, USA) in the dark for 30 min. Finally, the cells were washed and analyzed for cell cycle by FACSCalibur flow cytometry (BD, Franklin Lakes, NJ, USA).

### Wound healing assay

The NCOA5-silenced SW620 and NCOA5-overexpressed SW480 CRC cells and the corresponding control cells (1×10^6^ cells) were cultured in 6-well plates and grown to 70% confluence as a monolayer. The wound area was generated by scraping cells with a 10 μl pipette tip across the entire diameter of the well, and extensively rinsed with the fresh medium to remove all cellular debris. DMEM medium containing 2% FBS was then added to maintain cell growth during the experiment. Progression of tumor cell migration was observed and photographed at a low-power field (100×) under microscopy at the beginning and at 24 and 48 hours after wounding. The speed of closure reflected migratory ability of tumor cells.

### Transwell invasion assay

The NCOA5-silenced SW620 and NCOA5-overexpressed SW480 CRC cells and the corresponding control cells (5×10^5^ cells in 100 μl serum-free DMEM) were seeded onto the upper chamber of Transwell filters (8 μm pore size, Millipore, Billerica, MA, USA) which were precoated with Matrigel (Millipore, Billerica, MA, USA). The bottom chamber was filled with 500 μl DMEM medium containing 20% FBS. After 24 hours of incubation, the cells on the upper surface of filters were carefully swabbed. The cells on the lower filters were then fixed with 4% paraformaldehyde and stained by crystal violet solution. The number of invaded tumor cells was counted in 10 randomly selected fields at 200× magnification under microscopy.

### Xenograft mouse models

The female athymic BALB/c nude mice were subcutaneously (s.c.) injected with SW620-shNCOA5 3# vs SW620-shNTC, and SW480-LV-NCOA5 vs SW480-LV human CRC cells (2×10^6^ cells/mouse) (6 mice/group). Tumor growth *in vivo* was monitored by other investigators that were blinded to the group allocation. Tumor volume was measured with a caliper and calculated by the formula, tumor size=*ab*^2^/2, where *a* is the larger of the two dimensions and *b* is the smaller. The tumor-bearing mice were sacrificed 4 weeks after tumor cell inoculation and the xenografted tumors were then removed and weighted.

### Western blot analysis

The snap-frozen CRC and paired non-cancerous colorectal tissues were lysed in RIPA lysis buffer (Beyotime Biotechnology, Beijing, China) supplemented with a protease inhibitor cocktail (Roche Applied Science, Penzberg, Upper Bavaria, Germany) on ice for 60 min. The whole CRC cell lysates also harvested using RIPA lysis buffer with a protease inhibitor cocktail. After the protein concentrations were measured by using a bicinchoninic acid (BCA) protein assay kit (Thermo Fisher Scientific, Waltham, MA, USA), the lysates were loaded (30 μg/lane) and separated in 12% sodium dodecyl sulfate-polyacrylamide gel electrophoresis (SDS-PAGE) and transferred to polyvinylidene difluoride (PVDF) membranes (Millipore, Bedford, MA, USA). The membranes were blocked with 5% fat-free milk in PBS containing 0.1% Tween-20. The membranes transferred by tissue lysates were subsequently incubated with a primary antibody anti-NCOA5 (1:1000; Cat. No. A300-789A, Bethyl, Montgomery, TX, USA) or anti-GAPDH (1:3000; Cat. No. AP0063, Bioworld, St. Louis Park, MN, USA) (used as a loading control) at 4 °C overnight. The membranes transferred by cell lysates were incubated with primary antibodies including anti-NCOA5 (1:1000; Cat. No. A300-789A, Bethyl, Montgomery, TX, USA), anti-phospho-AKT (anti-p-AKT) (Ser473) (clone No. D9E) (1:1000; Cat. No. 4060S, Cell Signaling Technology, Danvers, MA, USA), anti-AKT (1:1000; Cat. No. AP52689-100, ABGENT, San Diego, CA, USA), anti-phospho-extracellular signal-regulated kinase 1/2 (anti-p-ERK1/2) (Thr202/Tyr204) (clone No. 20G11) (1:1000; Cat. No. 4376S, Cell Signaling Technology, Danvers, MA, USA), anti-ERK1/2 (clone No. 137F5) (1:1000; Cat. No. 4695S, Cell Signaling Technology, Danvers, MA, USA), anti-Cyclin D1 (clone No. 92G2) (1:1000; Cat. No. 2978S, Cell Signaling Technology, Danvers, MA, USA), anti-P27 (clone No. D69C12) (1:1000; Cat. No. 3686S, Cell Signaling Technology, Danvers, MA, USA), anti-MMP9 (clone No. 5G3) (1:1000; Cat. No. GTX60482, GeneTex, Irvine, CA, USA) and anti-GAPDH (1:3000; Cat. No. AP0063, Bioworld, St. Louis Park, MN, USA) (a loading control), respectively. The membranes were then incubated with corresponding HRP-labeled secondary antibodies (1:5000; Beyotime Biotechnology, Beijing, China) for 1 hour. Finally, an enhanced chemiluminescence (ECL) detection kit (Millipore, Bedford, MA, USA) was used to protein signaling detection and the protein bands were exposed onto X-ray films.

### Enzyme-linked immunosorbnent assay (ELISA)

The SW620-shNCOA5 2# or SW620-shNCOA5 3# vs SW620-shNTC, and SW480-LV-NCOA5 vs SW480-LV CRC cells were seeded at 5×10^5^ cells/2 mL culture medium/well into 6-well plates. 24 hours after incubation, the above cell culture supernatants were collected. The amount of secreted MMP9 was then determined by ELISA analysis using a human MMP9 ELISA assay kit (R&D Systems Inc., Minneapolis, MN, USA) according to company's protocols.

### Gelatin zymography analysis

The SW620-shNCOA5 2# or SW620-shNCOA5 3# vs SW620-shNTC, and SW480-LV-NCOA5 vs SW480-LV CRC cells were cultured in DMEM medium containing 10% FBS in 75 cm^2^ flasks. When they were in 70% confluence, the cells were washed with serum-free DMEM medium and cultured in serum-free DMEM medium overnight. The conditioned medium was collected and concentrated with YM-3 Centricon membranes (Millipore, Billerica, MA, USA) at 7000 g for 4 hours. Proteins (5 μg/lane) were loaded on 10% polyacrylamide gel containing 0.1% gelatin (Sigma, St Louis, MO, USA). After electrophoresis, the gel was washed with washing buffer and incubated in incubation buffer for 24 hours at 37 °C. Then the gel was stained with 0.5% Coomassie blue R250 and destained for analysis of MMP9 gelatinolytic activity.

### PI3K inhibition assay

The NCOA5-overexpressed SW480-LV-NCOA5 CRC cells were pretreated with PI3K inhibitor LY294002 (Selleck Chemicals, Houston, TX, USA) at different final concentrations (0, 5, 10 and 20 μM) for 6 hours, and then subjected to Western blot analysis of p-AKT, AKT, Cyclin D1, P27 and MMP9. Additionally, the SW480-LV-NCOA5 CRC cells were treated with LY294002 (20 μM). 6 hours after pretreatment, the cells were extensively washed and then subjected to CCK-8, migration and invasion assays as described above. The untreated SW480-LV-NCOA5 or SW480-LV cells were used as controls.

### Cyclin D1/MMP9 siRNA assay

The SW480-LV-NCOA5 CRC cells were transfected with 100 nM human Cyclin D1 siRNA (siCyclin D1) (Cell Signaling Technology, Danvers, MA, USA), MMP9 siRNA (siMMP9) (Santa Cruz Biotechnology, Santa Cruz, CA, USA) or control siRNA (sicontrol) (Cell Signaling Technology, Danvers, MA, USA) using HiPerFect transfection reagent (Qiagen, Shanghai, China) following manufacturer's instructions. After 24 hours of transfection, the siCyclin D1- or sicontrol-transfected SW480-LV-NCOA5 and the untransfected SW480-LV-NCOA5 or SW480-LV cells were then subjected to CCK-8 assay. The siMMP9- or sicontrol-transfected SW480-LV-NCOA5 and the untransfected SW480-LV-NCOA5 or SW480-LV cells were subjected to migration and invasion assays.

### Statistical analysis

All statistical analyses were carried out using SPSS13.0 software (SPSS Inc., Chicago, IL, USA) for Windows. All of the quantitative data were expressed as mean±standard deviation (SD). Statistical analyses were conducted with Pearson's χ^2^ test, Fisher's exact test, Student *t* test and one-way or two-way repeated measures analysis of variance (ANOVA). A value of *P*<0.05 was considered statistically significant.
